# Mechanism of Efficient Smithsonite Flotation with a Ternary Composite Collector Under Sulfur-Free Conditions

**DOI:** 10.3390/molecules29246014

**Published:** 2024-12-20

**Authors:** Rui Li, Yanhai Shao, Jinhui Li, Chenjie Liu, Hongqin Chen, Xiao Meng, Xinru Jia

**Affiliations:** Faculty of Land Resources Engineering, Kunming University of Science and Technology, Kunming 650093, China; 20222101118@stu.kust.edu.cn (R.L.); 15096956049@163.com (J.L.); 19967824778@163.com (C.L.); chq725309@163.com (H.C.); mengxiao20211202@163.com (X.M.); 13753493605@163.com (X.J.)

**Keywords:** flotation, smithsonite, composite collector, hydroxylation

## Abstract

The increasing demand for zinc resources and the declining availability of sulfide zinc ore reserves have made the efficient utilization of zinc oxide a topic of considerable interest. In this study, a ternary composite collector ABN (Al-BHA-NaOL system) was applied to the direct flotation of smithsonite. Micro-flotation studies showed that at pH 9, ABN exhibited better adsorption on smithsonite, achieving a recovery rate of 80.62%. Visual MINTEQ 3.1 and zeta potential analysis confirmed that ABN predominantly reacted with Zn(OH)_2_(aq) on the surface of smithsonite. Furthermore, X-ray photoelectron spectroscopy (XPS) analysis results elucidated the formation of Al-O bonds through chemical adsorption on the smithsonite surface. Additionally, powder contact angle measurements indicated that ABN enhances the surface contact angle of smithsonite. These results illuminate that ABN is adsorbed by reacting with O sites on hydroxylated metal ions on the smithsonite surface, with Al serving as the adsorption center, thereby achieving separation and purification. Due to ABN’s adsorption characteristics, smithsonite can achieve efficient and clean direct flotation recovery without sulfidization.

## 1. Introduction

Zinc metal resources primarily originate from sulfide zinc ore and oxide zinc ore. Due to the increasing demand for zinc metal and the relative depletion of sulfide zinc ore, efficient recovery of zinc from oxide zinc ore has become a crucial focus in the industry [1,2,3,4,5]. Smithsonite, a principal oxide zinc ore, is primarily recovered through methods such as sulfidation flotation and direct flotation [6]. Although the sulfidization–amine flotation method [7,8,9] has demonstrated effective separation and purification of smithsonite, its efficacy is notably influenced by the presence of flotation slimes and soluble salts [10,11]. Similarly, the sulfidization–xanthate flotation method generally requires heating, activation, and the use of pentyl xanthate. Moreover, strict control over the amount of sulfidization reagent and the sulfidization time is essential for effective flotation. Additionally, the flotation process involves the addition of significant amounts of Na_2_S and other alkaline agents, which results in the wastewater becoming highly alkaline. The introduction of xanthates, black medicines, and other reagents further exacerbates the problem, causing the dichromate index (CODcr) to significantly exceed acceptable standards. Residual collectors and their derivatives pose severe environmental risks, including the contamination of groundwater and soil. The health risks associated with flotation reagents to humans, animals, and aquatic life have been extensively reviewed in the literature [12,13]. Concerning the environmental impact of smithsonite sulfide flotation, there remains considerable potential for the development and improvement of vulcanization processes. Although many new vulcanizing agents offer environmental benefits and higher efficiency, they face challenges such as high costs, manufacturing complexity, and limited applicability in industrial operations.

In the direct flotation of smithsonite, polar collectors, including fatty acids and their derivatives, as well as hydroxamic acid collectors, are commonly employed. Fatty acids and their derivatives exhibit strong collecting efficacy yet demonstrate limited selectivity for smithsonite [14,15]. In contrast, the unique molecular structure of hydroxamic acid collectors allows for chelation with metal ions, altering the surface characteristics of the minerals and enhancing the adhesion between bubbles and minerals. Although hydroxamic acid exhibits superior selectivity in the flotation of smithsonite, its collecting efficacy is comparatively diminished [16,17,18,19,20]. Consequently, the quest for effective collectors to separate and purify smithsonite more accurately and efficiently is a key area of investigation in the realm of the direct flotation of smithsonite. Studies have demonstrated that a 2:1 molar ratio of sodium oleate (NaOL) to benzyl hydroxamic acid (BHA) significantly enhances the flotation effect [21,22]. This synergistic interaction reduces gas–water interfacial tension, increases the surface activity of minerals, and markedly improves the enrichment effect on smithsonite after sulfidation modification. However, the application of the binary compound reagent BHA/NaOL necessitates a sulfidation reaction on the minerals to achieve optimal flotation performance.

Researchers have explored the concept of chelating interactions between metal ions and organic compounds to assemble binary composite collectors. For example, the utilization of Al^3+^ in conjunction with BHA to create an Al-BHA chelate for the flotation of ilmenite demonstrates significantly superior efficacy compared to a sequential method where Al^3+^ is applied first, followed by BHA. The Al-BHA chelate forms a more stable Al-O bond on the mineral surface, leading to stronger adsorption of the chelate [23]. Despite this, the Al-BHA chelate maintains a considerable degree of unsaturation, which may lead to the formation of polymers and the consumption of reagents, thereby diminishing recovery rates [24].

Building on the Al-BHA and BHA/NaOL systems, a ternary composite collector, ABN, which consists of Al-BHA and NaOL, was developed [25]. This collector is designed to facilitate the clean and efficient flotation of smithsonite in a mildly alkaline environment. Direct flotation of smithsonite using ABN reduces the recovery conditions of smithsonite (lower pH and temperature conditions); due to the adsorption characteristics of ABN, sulfidization treatment of smithsonite is rendered unnecessary, thus simplifying the flotation process.

## 2. Result and Discussion

### 2.1. Micro-Flotation

**Figure 1:**
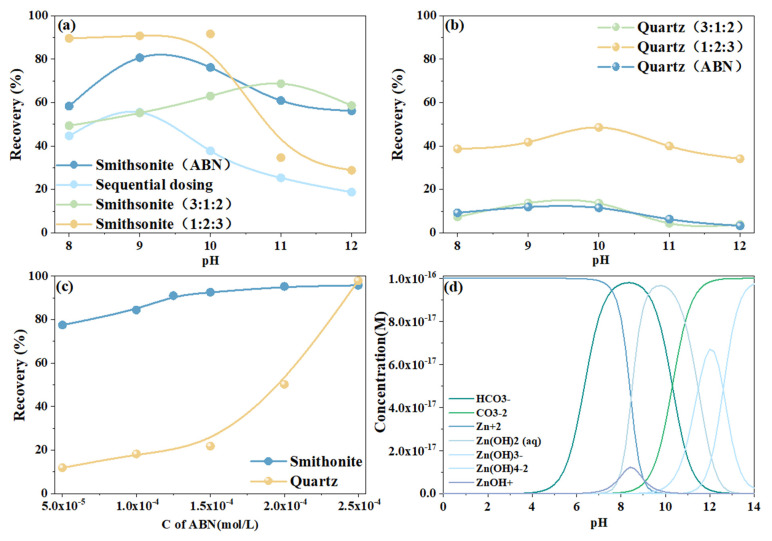
Recovery rate of smithsonite under different experimental condition (**a**) different reagent regimes recovery rate of smithsonite; (**b**) different reagent regimes recovery rate of quartz; (**c**) dosage experiment (**d**) composition diagrams of smithsonite solution under different pH by Visual MITEQ3.1.

To verify the separation characteristics and collectivity of ABN, and to determine its optimal conditions, flotation experiments on smithsonite and quartz were conducted. Figure 1a,b compare the flotation recovery rates of smithsonite and quartz under different pH values and reagent regimes (with a reagent concentration of 0.5 × 10^−4^ mol/L). When ABN (Al:BHA:NaOL, reagent molar ratio 2:3:1) was used, the recovery rate of smithsonite increased significantly with pH, with this trend continuing until it reached a peak, after which a gradual decline was observed between pH 9 and 10. At pH values above 10, the recovery rate decreased markedly. In comparison, the recovery rate of smithsonite using the sequential dosing method was consistently at least 14% lower than that with the ABN compound reagent, highlighting the superior effect of the compound reagent method in improving recovery rates. Additionally, the reagent’s enrichment and separation performance under different molar concentration ratios (with a fixed compound reagent concentration of 0.5 × 10^−4^ mol/L) were evaluated. When the Al-BHA-NaOL system was used at a ratio of 3:1:2, the reagent exhibited excellent separation characteristics, but the collectivity was relatively limited. Conversely, at a ratio of 1:2:3, the system showed enhanced collectivity but significantly reduced selectivity.

Figure 1c shows that under low-concentration conditions, the recovery difference between smithsonite and quartz was the greatest, indicating that ABN exhibits strong selectivity at low concentrations. Furthermore, as the ABN concentration increased, the recovery rate of gangue minerals increased significantly, with a noticeable upward trend at 1.5 × 10^−4^ mol/L. These results confirm that ABN demonstrates strong selectivity at low concentrations, while at higher concentrations, its collecting capacity is enhanced.

Prior studies have suggested that the ideal pH range for smithsonite flotation is between 10.5 and 12 [14,26]. However, this finding is inconsistent with the results obtained in the present experiments. Figure 1d presents the compositional diagrams of the smithsonite solution at different pH conditions. At pH 8, the main species in the solution were Zn^2^⁺ and HCO_3_^−^, with flotation recovery significantly lower than at pH 9. At pH 9, the primary species were Zn(OH)_2_(aq) and HCO_3_^−^, with a peak recovery observed, attributed to the formation of Zn(OH)_2_(aq), leading to a sharp increase in flotation recovery. This result aligns with previous studies [25]. ABN mainly reacts with the hydroxide species on the surface of smithsonite. Since the concentration of HCO_3_^−^ did not vary significantly between pH 8 and pH 9, and the presence of CO_3_^2−^ did not affect the flotation recovery increase during this period, it can be confirmed that HCO_3_^−^ and CO_3_^2−^ have no significant effect on the interaction between ABN and smithsonite. Between pH 9 and 10, the main species in the solution included Zn(OH)_2_(aq), CO_3_^2−^, and HCO_3_^−^, with most zinc existing in the form of hydroxide. This is because ABN binds to the oxygen sites of metal hydroxide ions on the surface of smithsonite, resulting in higher recovery rates at around pH 9. As the pH increased further, Zn(OH)_3_^−^ began to appear, and flotation performance gradually declined. At pH 11, Zn(OH)_4_^2−^ formed, corresponding to a sharp decline in flotation recovery.

In summary, the optimal flotation performance was achieved using ABN at a ratio of 2:3:1, particularly at a pH of 9. Furthermore, compared to the sequential dosing method, the ABN reagent exhibited superior efficiency in mineral enrichment. The reagent concentration also emerged as a critical factor, significantly influencing the differential flotation behavior of the minerals.

### 2.2. Zeta Potential Determination

**Figure 2:**
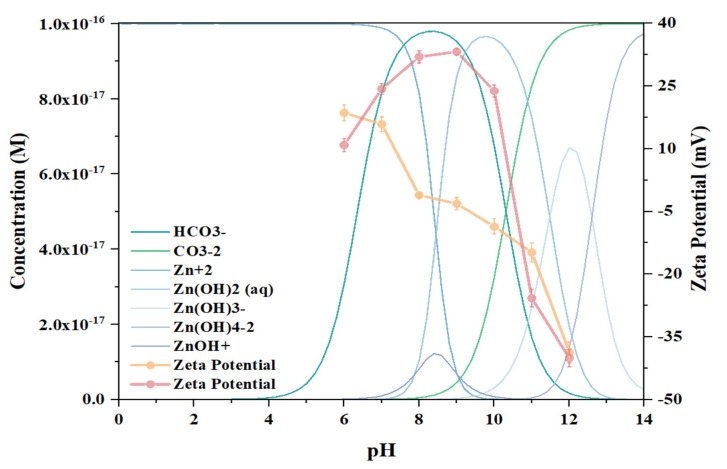
Effect of pH on the zeta potential and composition diagram of smithsonite solution by Visual MINTEQ analysis.

To further study the mechanism by which ABN interacts with smithsonite and to investigate the alterations in surface charge, measurements of the Zeta potential were conducted on both untreated smithsonite and smithsonite that had been treated with ABN, as shown in Figure 2.

The isoelectric point (IEP) of untreated smithsonite is approximately 7.9, aligning with findings from earlier research [27,28]. Notably, when the pH surpasses 10, there is a significant reduction in the Zeta potential as pH levels rise. This phenomenon can primarily be attributed to the dissolution characteristics of smithsonite, the formation of surface charges, and the hydrolysis reactions involving dissolved ions during the dissolution process, which collectively contribute to the observed trend [29,30].

In this study, ABN is a ternary composite collector that incorporates the metal cation Al^3^⁺. The Zeta potential trend in smithsonite treated with ABN differs from that observed in sulfidation flotation [31]. Initially, the Zeta potential increases with the rise in pH, but sharply decreases when pH exceeds 10. Between pH 7 and 10, Al^3^⁺ acts as the primary adsorption center for ABN, causing a significant increase in surface potential. However, at pH 11, Al^3^⁺ undergoes complete hydroxylation, preventing it from functioning as the adsorption center. In the pH range of 8 to 10, the changes in surface potential are more pronounced compared to untreated smithsonite, indicating significant ABN adsorption with Al^3^⁺ as the central ion. The adsorption peak occurs at pH 9, where the surface charge difference between treated and untreated smithsonite is maximal, suggesting that recovery is significantly enhanced at this optimal pH.

The solution composition diagram of smithsonite across different pH levels was analyzed and visualized using Visual MINTEQ 3.1. Notably, at a pH of 9, the primary species identified in the solution include Zn(OH)_2_(aq) and HCO_3_^−^. At this specific pH level, the system reaches an adsorption peak. The observation of this peak suggests that the adsorption behavior is significantly influenced by the hydroxylated forms of zinc present in the solution. Furthermore, it has been confirmed that the ABN predominantly interacts with the hydroxylated zinc species on the surface of the smithsonite. This chemical interaction plays a crucial role in the effective collection of the minerals.

When the pH level rises above 9, the solution begins to generate Zn(OH)_3_^−^. As the pH continues to increase, the concentration of this species also rises, peaking at a pH of 12. During this period, the Zeta potential experiences a decline as pH increases, and the presence of Zn(OH)_3_^−^ contributes to this reduction in the Zeta potential of smithsonite, thereby depressing the adsorption of ABN. At a pH level of 11, the metal ions and component Al^3+^ present on the surface of smithsonite undergo complete hydroxylation. In this specific pH environment, component Al^3+^ is unable to interact with the hydroxylated ions on the smithsonite surface, which limits its function as an adsorption center [21].

The adsorption of ABN [23] on the mineral surface primarily occurs through three mechanisms: single-component adsorption, binary complexation adsorption, and multivariate complexation adsorption. The benzene ring of BHA and the Carboxyl group of NaOL can form hydrogen bonds, resulting in high-molecular-weight complexes that adsorb onto the mineral surface.

At a pH of 6 or 11, the agents are mainly adsorbed through single-component adsorption (BHA; NaOL) or binary complexation adsorption (NaOL-BHA). Consequently, at these pH levels, the surface potential of smithsonite, after ABN treatment, becomes more negative.

### 2.3. X-Ray Photoelectron Spectroscopy (XPS) Analysis

**Figure 3:**
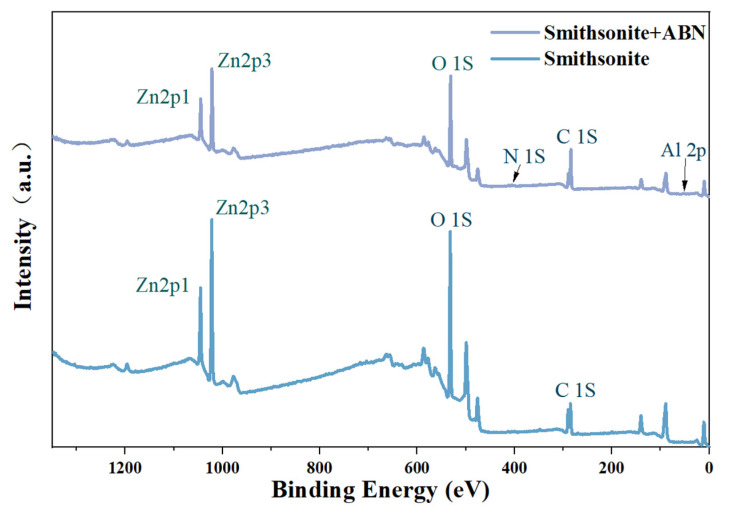
Full XPS spectrum of the smithsonite samples.

To investigate the surface material changes of smithsonite before and after ABN treatment, XPS analysis was performed, resulting in the acquisition of the XPS full spectrum and atomic concentration data, as shown in Figure 3 and Table 1, of smithsonite before and after ABN treatment. The figure illustrates a notable alteration in the intensity of the C 1s and O 1s peaks within the full spectrum following the ABN treatment. Additionally, the surface of the minerals exhibits faint peaks corresponding to N and Al. The data presented in Table 1 allow for the conclusion that, following ABN treatment, there was a reduction of 10.14% in the concentration of oxygen atoms on the surface of smithsonite, while the concentration of carbon atoms experienced an increase of 12.09%. The presence of trace amounts of nitrogen and aluminum atoms on the surface can be attributed to the components BHA and Al^3+^ found in the treatment agent, respectively, thereby providing evidence that ABN has been adsorbed onto the surface of smithsonite.

**Figure 4:**
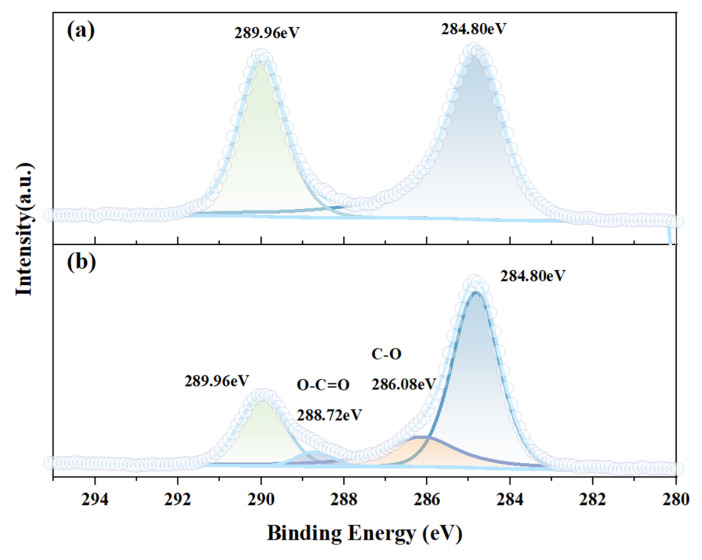
Fine spectrum C 1s spectra of the smithsonite surface under different conditions. (**a**) Smithsonite and (**b**) smithsonite + ABN.

The fine spectrum of smithsonite C 1s is illustrated in Figure 4, showcasing the results before (a) and after (b) the application of ABN treatment. In Figure 4a, the spectrum reveals two distinct peaks corresponding to the smithsonite sample that underwent treatment with deionized water at a pH of 9; these peaks represent carbon associated with the C-(C, H) bond at 284.80 eV and the carbonate ion (CO_3_^2−^) at 289.96 eV within the ZnCO_3_ structure. Conversely, in panel (b), the spectrum displays four peaks, including the previously mentioned carbon (284.80 eV) and carbonate (289.96 eV) signals, along with additional peaks for O-C=O at 288.72 eV and C-O at 286.08 eV, indicating the presence of new bonding configurations in the smithsonite sample treated with ABN under identical conditions. The O-C = O group comes from the carboxyl group of the oleic acid component (NaOL), and the C-O group comes from BHA and NaOL. BHA exhibits tautomeric forms characterized by the groups -C=N- or -C=O. Upon interaction with metal ions, BHA forms a chelate that result in a multi-ring configuration, wherein the five-membered ring structure demonstrates a greater degree of stability compared to other possible arrangements [32]. The emergence of the C-O bond at 285.44 eV, as illustrated in Figure 4b, substantiates the notion that ABN predominantly adheres to the mineral surface in a five-membered ring configuration throughout this process. The XPS results distinctly illuminate that both components, BHA and NaOL, within ABN are present on the surface of smithsonite.

**Figure 5:**
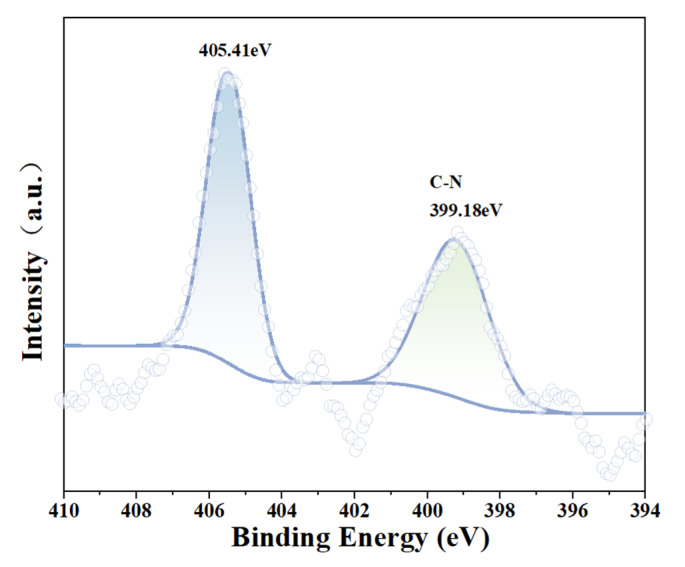
Fine spectrum N 1s spectra of the smithsonite surface after ABN treatment.

Figure 5 is the N 1s fine spectrum of smithsonite after ABN treatment. N is the characteristic component of the BHA agent. It can be clearly seen that C-N (399.18 eV) is produced on the mineral surface, which confirms that ABN is adsorbed on the mineral surface.

**Figure 6:**
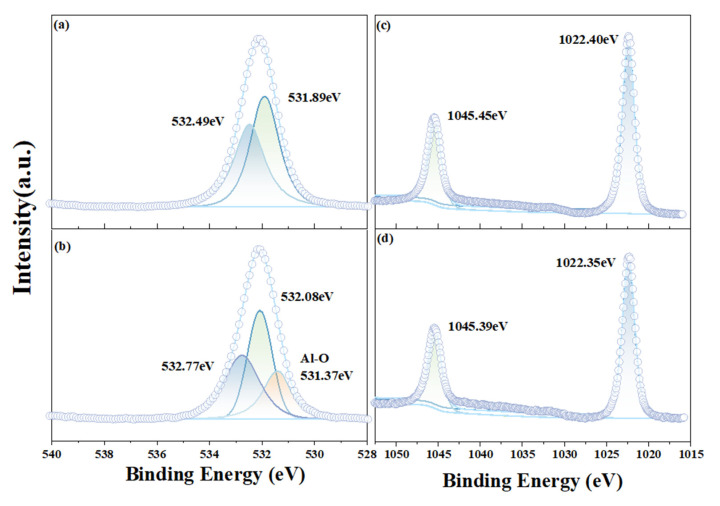
Fine spectrum of the smithsonite surface under different conditions; (**a**) O 1s smithsonite; (**b**) O 1s smithsonite + ABN; (**c**) Zn 2p smithsonite; (**d**) Zn 2p smithsonite + ABN.

Figure 6 is the fine spectra of smithsonite O 1s and Zn 2p before (a) (c) and after (b) (d) ABN treatment. The peaks of O 1s in (a) are 531.89 eV and 532.49 eV, respectively, representing the characteristic peaks of the Zn-O bond and -OH in smithsonite. From Figure 6b, it can be seen that the peak of O 1s increases by 0.19 eV and 0.28 eV, respectively, corresponding to the above two characteristic peaks (Zn-O bond and -OH) after ABN treatment, which proves that the surface chemical environment of smithsonite is changed. It is worth mentioning that the characteristic peak corresponding to the Al-O bond (531.37eV) was generated on the surface of smithsonite after ABN treatment, and with the addition of ABN, the relative area of the peak characterized by -OH decreased, and the binding energy intensity increased. It is proved that ABN and the hydroxylated metal ions on the surface of smithsonite interact, with O serving as the active site in this interaction. This interaction causes the dissociation of H⁺ ions and the formation of Al-O bonds. Through chemical adsorption, the adsorption sites on the smithsonite surface are connected to the metal ion complexes in ABN, and then the NaOL and BHA components in ABN are adsorbed on the mineral surface.

The data presented in (c) and (d) indicate that there is no significant change in the peak value of Zn 2p following ABN treatment, suggesting that ABN does not utilize the Zn site for adsorption. Instead, it appears that ABN employs the O bond as a connecting agent to interact with the Al site within the ABN structure.

### 2.4. Powder Contact Angle

**Figure 7:**
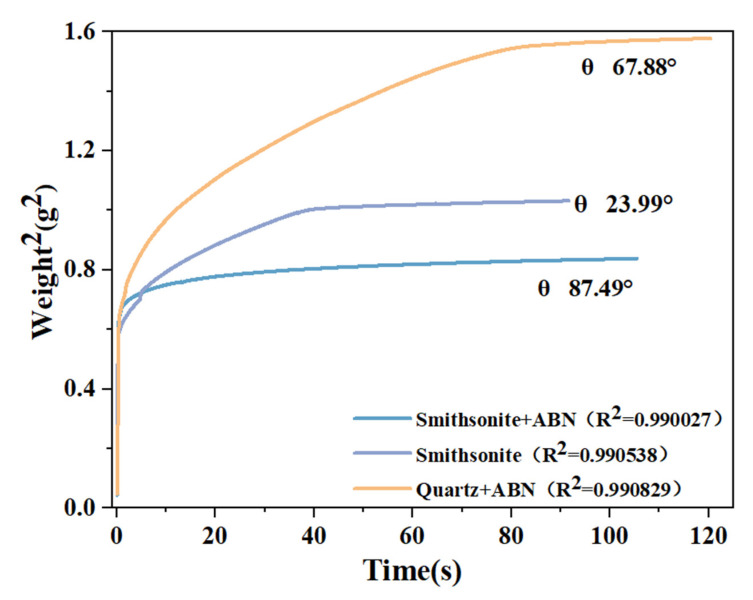
Powder contact angle test curve.

Figure 7 shows the contact angle and goodness-of-fit curves of smithsonite and quartz powder at pH 9. The goodness of fit (R^2^) of the samples was greater than 0.99. The contact angle of smithsonite powder after ABN treatment was 87.49°, which was 63.5° higher than that of untreated smithsonite (23.99°). The surface characteristics of smithsonite exhibit a greater degree of hydrophobicity, thereby indicating that ABN has the capacity to enhance the hydrophobic nature of smithsonite’s surface. In previous studies [33,34,35], it was found that untreated quartz, characterized by its hydrophilic nature, typically exhibits a contact angle of under 50 degrees. However, when the pH is adjusted to 9 and ABN is introduced, the contact angle of quartz increases to 64.88 degrees. In contrast, when comparing quartz to smithsonite under identical conditions, the contact angle of quartz remains relatively stable, showing no significant variation.

The findings from the contact angle measurements elucidated that ABN exhibited a pronounced selectivity, with chemical adsorption identified as the primary mechanism of action at a pH of 9. The lack of hydroxylated metal ions on the quartz surface prevents ABN from undergoing chemical adsorption onto mineral surfaces, resulting in a minor alteration in the wettability of quartz when contrasted with that of smithsonite.

### 2.5. Discussion

Numerous previous studies have demonstrated that the optimal flotation conditions for smithsonite occur within the pH range of 10.5 to 12. However, in this study, the recovery was the highest at a pH of 9. This is because most of the Zn in the solution’s chemical system exists in hydroxylated forms. It has previously been confirmed that the adsorption of ABN exists in multiple forms (single-component adsorption, binary complexation adsorption, and multivariate complexation adsorption), with chemical adsorption being confirmed as the primary form. A limited quantity of ABN exhibits enhanced collectivity and selectivity for target minerals. Excessive ABN enhances its mineral collectivity but compromises its selectivity. This effect is primarily due to the increased reagent concentration, particularly the strong collectivity of NaOL, which directly adsorbs onto the mineral surface. The elevated concentration promotes diverse adsorption modes on the mineral surface, leading to excessive adsorption on gangue minerals and reducing overall selectivity. In addition, ABN can significantly improve the hydrophobicity of the mineral surface.

**Figure 8:**
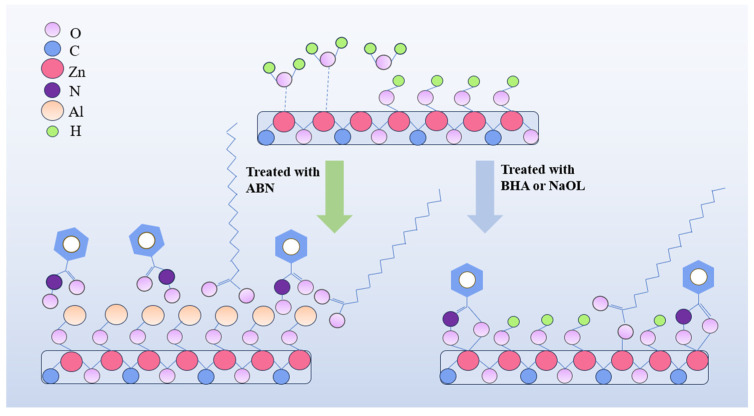
Mechanism diagram of ABN on smithsonite surface.

The trends in XPS and Zeta potential indicate that ABN preferentially adsorbs onto the mineral surface through Al^3+^, with the adsorption peak appearing at a pH of 9. The formation of Al-O bonds on the mineral surface indicates that chemical adsorption has occurred. Further confirmation, using a combination of Visual MINTEQ 3.1 software and Zeta potential changes, reveals that ABN primarily reacts with hydroxylated metal ions on the smithsonite surface. Al^3+^ serves as the adsorption center, interacting with hydroxyl ions on the smithsonite surface (Figure 8).

Previous research has not extensively investigated the direct flotation of smithsonite, and the sulfidation flotation of this mineral has encountered certain limitations. However, within the Al-BHA-NaOL system, this investigation revealed that the employment of a ternary composite collector, ABN, can significantly enhance the direct flotation process for smithsonite. ABN interacts with the hydroxylated metal ions on the surface of smithsonite by utilizing Al^3+^ as an adsorption center, leading to the disruption of OH bonds and the release of H⁺ ions, resulting in the formation of new Al-O bonds. This interaction effectively separates and purifies smithsonite. Compared with the traditional sulfidation flotation system, ABN direct flotation reduces the recovery conditions (pH and temperature) of smithsonite and achieves efficient recovery through a simpler process. Since ABN mainly reacts with the hydroxylated metal ions on the surface of smithsonite, the sulfidation process is unnecessary, which reduces reagent usage and the difficulty of industrial water treatment and presents a more environmentally friendly alternative.

Recently, ABN has been demonstrated as an effective reagent for the clean and efficient flotation of ilmenite. Its cost, molecular configuration, reagent stability, and implications for beneficiation wastewater have been evaluated under industrial conditions [23,24,25]. This investigation delineates the mechanism by which ABN promotes the direct flotation of smithsonite, while acknowledging that its practical application demands further investigation, particularly in industrial environments. The findings reveal that the recovery rate of smithsonite using the ABN system is significantly higher compared to traditional sulfidation flotation techniques [36,37,38].

In the flotation process, in order to reduce production costs, conserve water resources, and comply with environmental policies, companies often need to adopt wastewater recycling. In conventional sulfidation flotation, if the residual flotation reagents are not effectively removed, this can lead to decreased separation efficiency, especially in the case of complex polymetallic ore processing plants containing both oxidized and sulfide ores. Moreover, sulfur-containing organic wastewater poses significant risks to the environment, humans, and animals [39,40], making its treatment relatively challenging. However, wastewater treated with ABN contains a small amount of polymeric aluminum salts, which can be recovered and used for subsequent applications. These aluminum salts may also serve as selective adsorbents for phosphates [41,42,43]. The residual NaOL [44,45] and BHA [46] components in the wastewater are relatively simple to treat.

Future inquiries may explore the implementation of smithsonite production within this system. Current evidence suggests that the use of the ternary composite collector ABN for direct smithsonite flotation is a viable option, offering environmental advantages over sulfidation flotation by simplifying the flotation process. This innovative approach highlights ongoing efforts to optimize zinc extraction processes, addressing the challenges posed by traditional methods while fostering advancements in flotation technology.

## 3. Experimental Section

### 3.1. Materials and Reagents

#### 3.1.1. Materials

Smithsonite samples (from Lanping County, Nujiang City, Yunnan Province, with a ZnCO_3_ content of 98%) and quartz samples (from Hunan, China, with a SiO_2_ content of 99.19%) were separated and purified using physical methods. Initially, the ore lumps were manually crushed, followed by fine pulverization using an agate mortar and pestle. After comminution, the pure mineral samples were sifted through standard mesh screens, ensuring that both samples met the required test standards. For micro-flotation tests, particles sized −74 + 38 μm were utilized, while particles −37 μm were selected for further detection and analysis.

**Figure 9:**
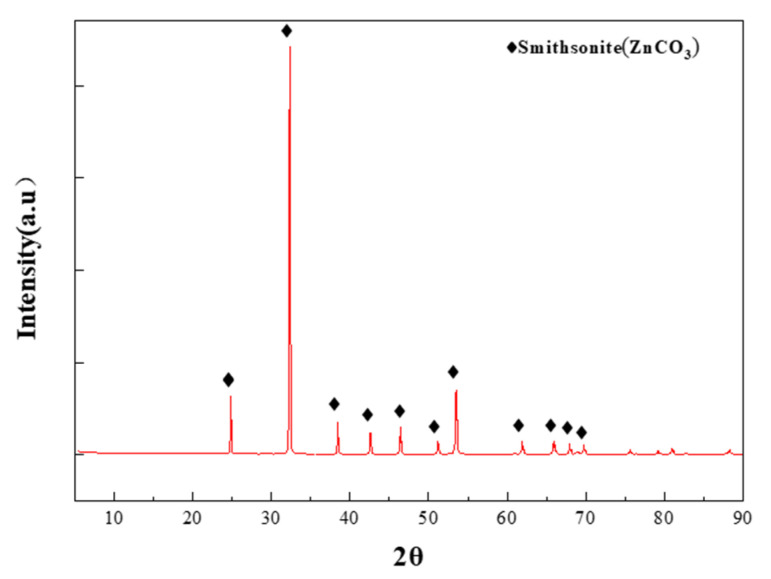
XRD spectrum of pure smithsonite.

XRD analysis was conducted to assess the purity of the mineral samples, and the results are presented in Figure 9. The findings indicate that the crystalline substances corresponding to each diffraction peak are primarily smithsonite (Figure 9).

#### 3.1.2. Reagents

The ternary composite collector was synthesized utilizing analytically pure aluminum chloride (AlCl_3_), benzohydroxamic acid (BHA), and sodium oleate (NaOL) in a molar ratio of 2:3:1 [25]. All reagents were obtained from Aladdin Reagent Co., Ltd. (Shanghai, China). Deionized water with a resistivity of 18.2 MΩ·cm was used for all experiments.

#### 3.1.3. Pre-Formation of Al-BHA-NaOL Complexes

An appropriate amount of solid reagent was mixed with 50 mL of deionized water to prepare a 1 × 10^−3^ mol/L solution. Based on the molar concentration ratio of the reagents, a specified amount of solution was taken and stirred under ambient temperature and pressure for 10 min to form a milky white solution, constituting the ABN ternary composite collector.

### 3.2. Micro-Flotation

The micro-flotation tests were conducted using an XFG II 5–35 g hanging slot flotation machine (Jilin Jitan Machinery Co., Ltd., Changchun city, Jilin province, China), equipped with a 40 mL effective volume organic glass flotation cell, operating at a rotation speed of 1800 r/min. All experiments were conducted at room temperature (17 °C).

**Figure 10:**
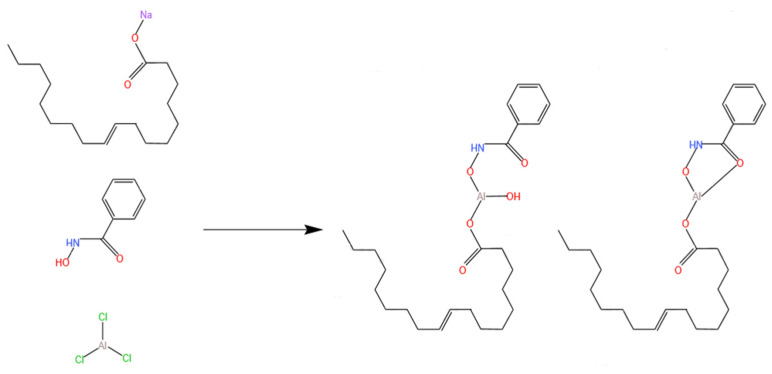
Chemical structure diagram of ABN.

The test process: 2 g of the sample and 38 mL of deionized water were added to a 40 mL micro-flotation cell and mixed for 3 min, during which the pH of the pulp was adjusted. ABN was then introduced and allowed to interact for 3 min. Subsequently, 2 μL of pine oil was added, with a reaction time of 1 min. Finally, the froth products and tailings were filtered and dried. The test procedure is illustrated in Figure 10.

**Figure 11:**
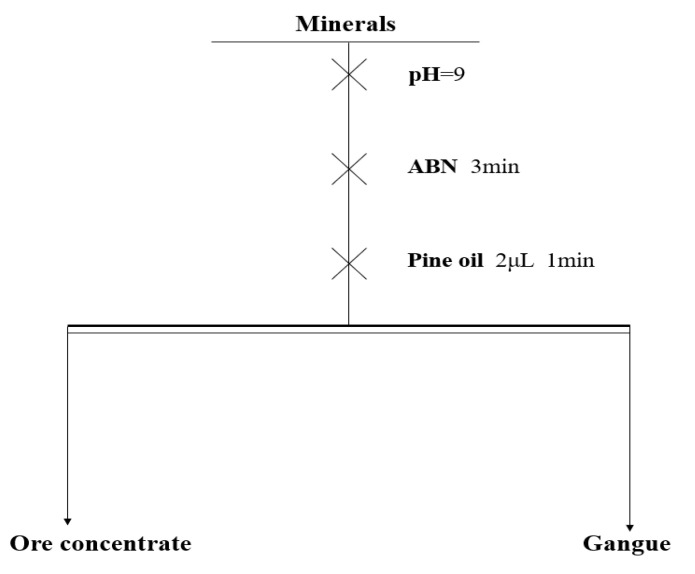
Flotation flowsheet.

To verify the difference between using the ABN reagent and the sequential dosing method, a sequential dosing experiment was conducted as follows: 2 g of the sample was added to a 40 mL micro-flotation cell, followed by 38 mL of deionized water, and then the mixture was stirred for 3 min with pH adjustment as needed. AlCl_3_, BHA, and NaOL were then added sequentially, allowing each reagent to interact with the mineral for 3 min before the next addition. Finally, 2 μL of pine oil was introduced. The froth products and tailings were filtered and then dried. The dosages of reagents used corresponded to the ABN reagent concentration ratios (Figure 11).

### 3.3. Zeta Potential Determination

A Malvern Zetasizer Nano ZS90 (Malvern Instrument, Malvern, UK) was used to measure the Zeta potential of the smithsonite surface. A sample of 50 mg of smithsonite was collected for each trial, followed by the addition of 50 mL of a 0.1% KCl solution. The pH of the resulting slurry was modified by incorporating either H_2_SO_4_ or NaOH, targeting a pH range of 6 to 12. Subsequently, ABN was introduced into the mixture and stirred for a duration of five minutes using a magnetic stirrer, after which the mixture was allowed to stand for an additional five minutes. The supernatant was then extracted for the purpose of determining the Zeta potential, with each sample being measured a minimum of three times to ensure an accurate average [47,48]. The Zeta potential test results were combined with Visual MINTEQ3.1 for analysis.

### 3.4. X-Ray Photoelectron Spectroscopy (XPS) Analysis

The smithsonite samples and ABN reaction samples were analyzed by XPS. An amount of 2 g of minerals with particle size of 37 μm was placed in a 50 mL beaker, the pH was adjusted to 9, ABN was added to react for 3 min, and then the samples were filtered, rinsed three times with deionized water, and dried in a vacuum oven. The XPS analysis equipment used for the dried samples was a Thermo Scientific K-Alpha (Waltham, MA, USA). The analysis of X-ray photoelectron spectroscopy results was conducted utilizing the AVANTAGE (5.9931.0.6755) software, with calibration performed against the standard binding energy of C-C, which is established at 284.80 eV [49].

### 3.5. Powder Contact Angle

The effective separation of flotation minerals is influenced by the wetting properties inherent to the minerals, with the contact angle serving as a critical parameter for characterizing these properties [50]. The principle and methodology for determining the contact angle involved the application of the Washburn method, utilizing a tensiometer-K100 (KR.U. SS, Hamburg, Germany) for measurement, while the resulting data were subsequently analyzed using ADVANCE software (https://www.kruss-scientific.com/zh-CN/products-services/advance-software/advance-drop-shape, accessed on 17 December 2024).

Samples were prepared under controlled conditions with a pH of 9, including three groups: pure smithsonite, smithsonite with 0.5 mL of ABN, and quartz with 0.5 mL of ABN. Each sample group was meticulously weighed to ensure a minimum mass of 6 g. Following preparation, the contact angles of the samples were measured. During the determination process, 2 g of samples was taken each time for one capillary constant measurement and one contact angle measurement. Anhydrous ethanol was used as the wetting liquid for capillary constant measurement, and deionized water was used as the wetting liquid for contact angle measurement [51].

The measurement calculation formula is as follows:

(1) $$m^{2}=\frac{c\rho^{2}\sigma \cos\theta }{\eta }t$$

(2) $$k=\frac{c\rho^{2}\sigma \cos\theta }{\eta }$$

(3) $$\theta =\arccos\frac{k\eta }{c\rho^{2}\sigma }$$

*m* represents the mass of the liquid that ascends within the tube due to capillary action (g), while *c* denotes the capillary constant of the powder (mm5). The variable *ρ* indicates the density of the liquid (g/mL), *σ* refers to the surface tension of the liquid (mN/m), and *η* signifies the viscosity of the liquid (mPa s). Additionally, *t* stands for time (s), *k* is the gradient of the linear relationship between *m*^2^ and time *t*, and *θ* represents the contact angle (°).

## 4. Conclusions

In this study, the ternary composite collector ABN, which was employed to directly float sphalerite, achieved remarkable flotation recovery in a weak alkaline environment.

1. The ternary composite collector ABN exhibits a pronounced adsorption effect on smithsonite, primarily through chemical adsorption. Al^3+^, serving as the adsorption center in the ternary composite collector, can be specifically adsorbed onto the surface of smithsonite, demonstrating absolute specificity.
2. At pH = 9, ABN exhibits superior selectivity and collectivity, demonstrating a stronger affinity for the target mineral. The use of compound agents proves to be significantly more effective than the sequential dosing method, enhancing overall flotation performance.
3. As the adsorption center of ABN, Al is adsorbed onto smithsonite’s surface by reacting with hydroxylated metal ions to form an Al-O bond. Consequently, its mechanism of action differs from that of sulfidation flotation.

Current research findings elucidate that using the ternary composite collector ABN for the direct flotation of smithsonite offers greater advantages over conventional sulfidation flotation. In mineral separation and purification, it exhibits strong absolute specificity. In terms of application, direct flotation using ABN is more environmentally friendly.

## Figures and Tables

**Table 1:** Relative concentration of elements on the smithsonite surface.

Sample	Assignment	C 1s	O 1s	Zn 2p	N 1s	Al 2p
Smithsonite	Atomic concentration	38.70%	49.2%	12.00%	-	-
Smithsonite + ABN	Atomic concentration	50.09%	39.41%	8.95%	1.03%	0.52%

## Data Availability

Data are contained within the article.
